# From healing to taking care

**DOI:** 10.1186/1824-7288-41-S2-A78

**Published:** 2015-09-30

**Authors:** Giuseppe Zampino

**Affiliations:** 1 Rare Diseases and Birth Defects Unit, Pediatric Department, Policlinico Universitario “A. Gemelli”, Università Cattolica del Sacro Cuore, Rome, Italy

## 

Disease is an absolute evil, the sick human being is an absolute good.

As doctors we are used to fighting disease to save the patient. But when disease and patient coincide, the picture changes. We must cure symptoms, prevent complications, reduce effects, but at same time we must help the family to accept their child. It is necessary to modify the approach moving from healing to caring.

Caring for the child is conditioned by recognition of his needs, that is identifying the concrete situation of the child and his family, in relation to his condition and its impact on their daily life.

The disabled child needs preventive and curative assistance, meaning a pediatric approach. In spite of big number of rare conditions, it is possible to sum up the pathologies which most frequently involve children with congenital but also acquired disabilities. The complexity of the assistance is due especially to the involvement of numerous strictly connected systems, therefore multidisciplinary and well coordinated approach is necessary. The coexistence of medical problems together with psychological and social aspects obliges the pediatrician to have a multisectorial view. The family pediatrician should use health balance to reveal the clinical problems frequent in a specific disability in order to avoid the most common complications of that condition, and to verify if the care project developed by social and medical operators is sustainable for the child, the family and the Country. The role of the hospital pediatrician consists in coordinating the interventions of different specialists, managing the emergencies and developing the treatment strategies calibrated for every single child and family [1]. So the correct caring of the child with malformative syndrome and more general with disability, includes an effective and functional integration among the internal clinical aspects, rehabilitative and educative aspects and psycosocial elements [2].

The advantage of coordinated and integrated assistance is clear also in economically terms, family satisfaction, and the team's cultural improvement.

The cost of an integrated assistance is compensated by a notable saving that the healthy system has for the cost reduction due to prevention of complications and to unnecessary exams [3].

Care means, first of all, giving high value to human relationships and taking into account the globality of the person, his needs and his problems. Always ensuring hope. The goal is to find equilibrium and sostenibility for the child and his family [4].
